# Establishment and Applicability of a Diagnostic System for Advanced Gastric Cancer T Staging Based on a Faster Region-Based Convolutional Neural Network

**DOI:** 10.3389/fonc.2020.01238

**Published:** 2020-07-28

**Authors:** Longbo Zheng, Xunying Zhang, Jilin Hu, Yuan Gao, Xianxiang Zhang, Maoshen Zhang, Shuai Li, Xiaoming Zhou, Tianye Niu, Yun Lu, Dongsheng Wang

**Affiliations:** ^1^Department of Gastroenterology Surgery, Affiliated Hospital of Qingdao University, Qingdao, China; ^2^State Key Laboratory of Virtual Reality Technology and Systems, Beihang University, Beijing, China; ^3^Department of Radiology, Affiliated Hospital of Qingdao University, Qingdao, China; ^4^Nuclear and Radiological Engineering and Medical Physics Programs, Woodruff School of Mechanical Engineering, Georgia Institute of Technology, Atlanta, GA, United States; ^5^Shandong Key Laboratory of Digital Medicine and Computer Assisted Surgery, Qingdao, China

**Keywords:** convolutional neural network, advanced gastric cancer, T staging, faster RCNN, artificial intelligence

## Abstract

**Background:** The accurate prediction of the tumor infiltration depth in the gastric wall based on enhanced CT images of gastric cancer is crucial for screening gastric cancer diseases and formulating treatment plans. Convolutional neural networks perform well in image segmentation. In this study, a convolutional neural network was used to construct a framework for automatic tumor recognition based on enhanced CT images of gastric cancer for the identification of lesion areas and the analysis and prediction of T staging of gastric cancer.

**Methods:** Enhanced CT venous phase images of 225 patients with advanced gastric cancer from January 2017 to June 2018 were retrospectively collected. Ftable LabelImg software was used to identify the cancerous areas consistent with the postoperative pathological T stage. The training set images were enhanced to train the Faster RCNN detection model. Finally, the accuracy, specificity, recall rate, F1 index, ROC curve, and AUC were used to quantify the classification performance of T staging on this system.

**Results:** The AUC of the Faster RCNN operating system was 0.93, and the recognition accuracies for T2, T3, and T4 were 90, 93, and 95%, respectively. The time required to automatically recognize a single image was 0.2 s, while the interpretation time of an imaging expert was ~10 s.

**Conclusion:** In enhanced CT images of gastric cancer before treatment, the application of Faster RCNN to diagnosis the T stage of gastric cancer has high accuracy and feasibility.

## Introduction

**Figure 1:**
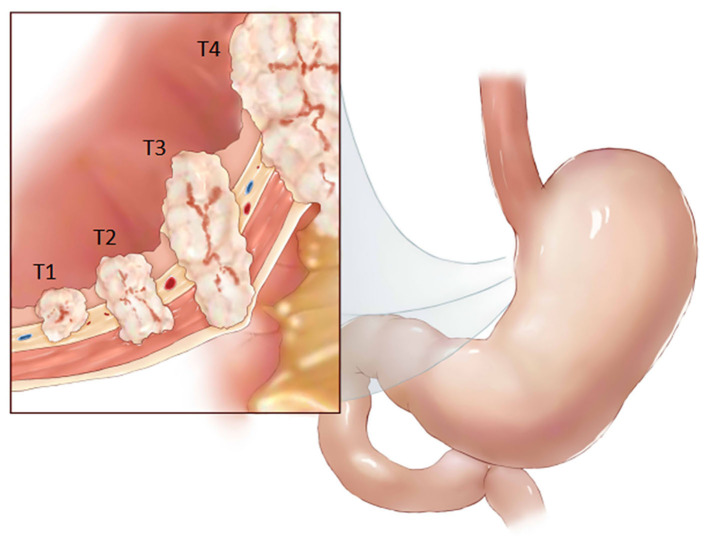
T stage pattern of gastric cancer.

Gastric cancer is currently ranked fifth in the global cancer incidence rate, and its mortality rate ranks third. Its high morbidity and mortality rates indicate a serious threat to human health (1). Tumor, node, and metastasis (TNM) stage and histological subtype is routinely used for risk stratification and treatment decision-making. For certain stage II and stage III, adjuvant chemotherapy is recommended as a standard preoperative treatment (2). Accurate preoperative T gastric cancer staging is critical for selecting treatment plans and predicting postoperative outcomes (T stage pattern of gastric cancer, see Figure 1). Therefore, early diagnosis and accurate staging before surgery are key to improving the accurate and prognosis. Because computed tomography (CT) is noninvasive, practical, convenient, and stable, it is a routine method used for the preoperative staging of gastric cancer (3). The application of abdominal enhanced CT greatly improves the accuracy of gastric cancer staging, and its accuracies of preoperative T staging and N staging are 62–75% and 75–80%, respectively (4–7). However, the final interpretation of a CT image still depends on the clinical experience and personal opinion of radiologists. Current research shows that texture analysis of CT images can be used to detect subtle differences that are unrecognizable by the human eye and that quantitative information regarding tumor heterogeneity can be obtained by analyzing the pixel intensity distribution and strength in images, thereby improving the diagnostic value of CT (8).

Artificial intelligence-mediated data processing has fast calculation speeds and high precision. Recently, convolutional neural networks (CNNs) have been increasingly used in clinical practice to evaluate medical images. This technique has been shown to have high diagnostic performance in diagnostic imaging, such as for coronary computed tomography angiography (CTA) (9) and X-ray detection in breast cancer (10). CNNs are the most mature algorithms among various deep learning models. This study was based on the powerful ability of CNNs to process and recognize images. We established a CNN-based clinical diagnostic system for progressive gastric cancer T staging utilizing preoperative abdominal enhanced CT images and verified and evaluated its accuracy. Artificial intelligence can be used to assist radiologists in the clinical T staging of gastric cancer based on preoperative abdominal CT. The process for establishing the system and the learning results are provided in this report.

## Methods

### Patients

**Figure 2:**
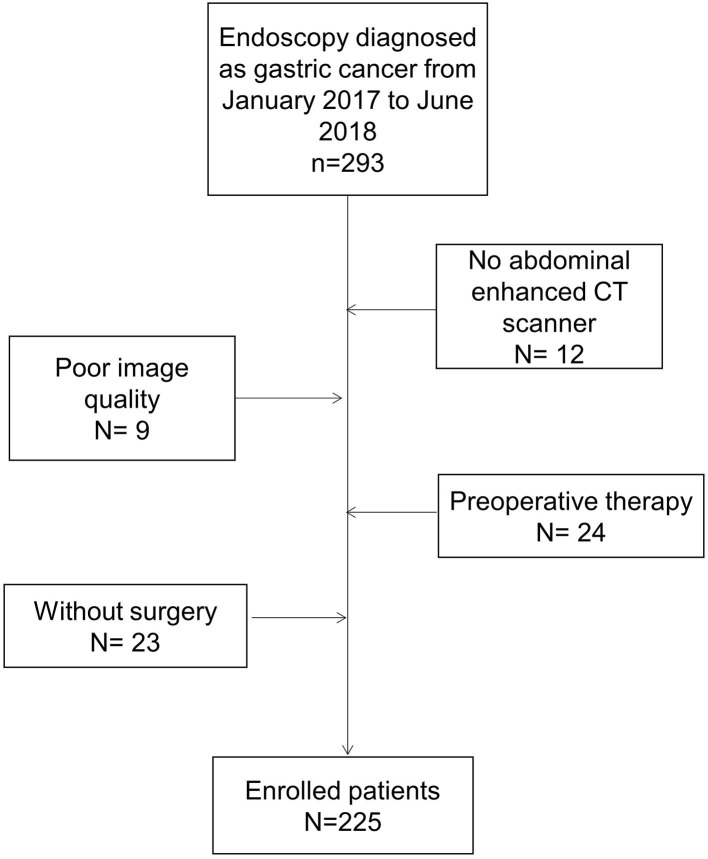
Flowchart of participant selection with exclusion criteria.

Our research team retrospectively collected data from 225 cases of advanced gastric cancer treated at the Affiliated Hospital of Qingdao University, China, from January 2017 to June 2018 (Figure 2). The study was approved by the Ethics Committee of the Affiliated Hospital of Qingdao University. All patients signed an informed consent form for the application of iodine contrast agent. The inclusion criteria were as follows: patients who underwent gastroscopy before surgery, were diagnosed with gastric cancer via pathological diagnosis and were not diagnosed with tumors in other areas; patients who underwent a preoperative upper abdominal enhanced CT examination in our hospital; and patients who underwent radical gastric cancer resection in our hospital, from which postoperative pathology led to a diagnosis of advanced gastric cancer.

We excluded patients if the primary tumor could not be identified on CT, or if patients had previously received adjuvant chemotherapy.

### Image Acquisition

A total of 225 patients were enrolled in this study. Basic patient information is provided in Table 1. Upper abdominal enhanced CT is a routine auxiliary examination for patients with gastric cancer and is used to screen patients who are highly suspected of having gastric cancer. Venous phase-enhanced CT imaging of the upper abdomen is superior to arterial phase-enhanced CT imaging for the diagnosis of gastric tumor infiltration; therefore, this study chose venous phase-enhanced CT images of the upper abdomen (11). A total of 3,500 images of advanced gastric cancer were obtained, including 990 T2 images, 1,134 T3 images, and 1,376 T4 images. All patients in this study underwent an upper abdominal enhanced CT scan using a Philips Brilliance iCT scanner. The scanning parameters included a layer thickness of 1 mm, an interlayer distance of 1 mm, and a spacing of 0.985. The patients had fasted for 4–6 h before the scan. Twenty minutes prior to the scan, the patients were given 500–1,000 ml of drinking water. For enhanced scanning, 90 ml of the nonionic contrast agent iohexol was injected into the antecubital vein at a rate of 3 ml/s using a high-pressure syringe. The scanning procedure was carried out as follows: after the injection of the intravenous contrast agent, there was a delay of 33 s in the arterial phase, 65 s in the venous phase, and 120 s in the equilibrium phase. The scan range was from the diaphragm to the umbilical plane.

**Table 1:** Clinicopathologic characteristics of patients in the cohorts.

**Variable**		**No**.	**%**	**Mean ± SD**
Age (year)	<60 years	116	51.56	59.70 ± 10.01
	≥60 years	109	48.44	
Gender	Male	153	68.00	
	Female	72	32.00	
Tumor size (cm)	<4	110	48.89	
	≥4	115	51.11	
Tumor differentiation	Well	9	4.00	
	Moderate	73	32.44	
	Poor	143	63.56	
Lauren type	Intestinal type	73	32.44	
	Diffuse type	77	34.22	
	Mixed type	75	33.33	
Depth of invasion	T2	64	28.44	
	T3	72	32.00	
	T4	89	39.56	
Lymph node metastasis	N0	90	40.00	
	N1	35	15.56	
	N2	42	18.67	
	N3a	46	20.44	
	N3b	12	5.33	
Tumor location	Cardia	16	7.11	
	Body	54	24.00	
	Antrum	155	68.89	
CA19-9 (U/ml)	Normal	210	93.33	21.27 ± 63.11
	Elevate	15	6.67	
CA72-4 (U/ml)	Normal	186	82.67	4.64 ± 11.93
	Elevate	39	17.33	
CEA (ng/ml)	Normal	203	90.22	2.84 ± 4.48
	Elevate	22	9.78	

*p, pathological*.

### Image Identification and Data Enhancement

We used the labeling software LabelImg to identify and label the images. Two senior radiologists independently interpreted the CT images and labeled the tumor lesions. The tumor segmentation method was used for labeling. According to the relevant literature, compared with the adjacent stomach wall, focal gastric wall thickening ≥ 6 mm was identified as abnormal thickening and canceration (4). Because the purpose of this study was to accurately identify the T stage of advanced gastric cancer, not to assess the detection ability of upper abdominal enhanced CT, the two radiologists only labeled the deepest position that the gastric lesion infiltrated into the stomach wall in the images, taking into consideration the patient's gastroscopy report and the final pathological results after surgery. According to the postoperative pathological results, a third radiologist examined the labeled tumor site on the upper abdominal enhanced CT images to ensure the accuracy and consistency of the lesions in the two enhanced CT images.

We used a data augmentation model to extract different regions of interest (ROIs) on the upper abdominal enhanced CT images and then used cropping, flipping, and other data enhancement methods to obtain additional images. This method enhanced the research data set while reducing the overfitting problem that could arise when the model processed the data set (12). After effective data augmentation, 5,855 advanced gastric cancer images were obtained.

### Faster R-CNN Principles and Training Processes

**Figure 3:**
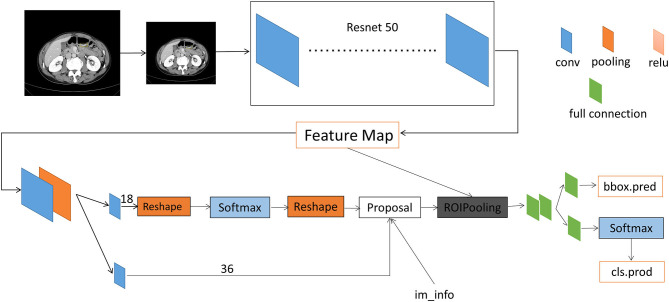
Architecture of faster region-based convolutional neural networks.

The automatic detection of T stage in gastric cancer images using a Faster R-CNN was studied (Supplementary Methods). The methods included a region proposal network (RPN) and the Fast R-CNN (Figure 3). In this experiment, RPN and Fast R-CNN were alternately trained in two stages, and the parameters were listed in Table 2.

**Table 2:** Training parameters of RCNN.

**No**.	**Parameters**	**Options**
1	Category number	4
2	Identify the category	Background, T2, T3, T4
3	Number of iterations	12,000
4	GPU ID	0
5	Base lr	0.0002
6	Display	20
7	Lr policy	Step
8	Gamma	0.01
9	Momentum	0.9
10	Weight decay	0.0005
11	Step size	90,000 and 30,000
12	Score threshold	0.05
13	NMS threshold	0.7

*Lr, learning rate; GPU ID, graphic processing unit identity document; NMS, Non-maximum suppression*.

Prior to establishing the diagnostic system, we pretreated the images, which included normalization of the image intensity range and image processing by histogram equalization (13). We uniformly scaled the images to 512 × 557 and then used a random sampling method to divide patients and their labeled images of various stages of advanced gastric cancer into a training set and a verification set in a ratio of 4:1; the consistency of the grouping was ensured. The images in the training set were then input into the diagnostic network for training. The skeleton of the CNN used in this study was a 50-layer deep CNN that could extract image features. Each level of the model was trained by 600 epochs. The stochastic gradient descent (SGD) optimizer was used with an initial learning rate of 0.0002.

### Faster R-CNN Database Validation Experiment

The classification performance of the system was verified using a verification set consisting of a subset of the same original data set. We conducted comparisons of the gastric cancer tumor area labeled by the radiologists with that by the system and determined the accuracy of the classification results for the verification set. A ROC curve was used to evaluate the area under the curve (AUC) of the CNN system for image accuracy, and the sensitivity, specificity, positive predictive value, and negative predictive value were determined. The flow chart of the test platform is shown in Figure 3.

### Statistical Analysis

The count data were descriptively analyzed using the number of cases (n) and percentage (%). According to the results of the Kolmogorov–Smirnov test, measurement data with a normal distribution were subjected to descriptive analysis using the mean ± standard deviation (mean ± SD). SPSS 20.0 software (SPSS, Chicago, IL) was used for statistical analysis. The computation learning results were analyzed using the Python programming language. Classification-report in the Metric module was used to generate multiclass conclusions, and the accuracy, recall, F1-score, and overall micro average, macro average, and weighted average in each class were recorded. The numbers of true positives and false positives at all nodes were counted, and the true positive rate and false positive rate under different probability thresholds were calculated to plot the ROC curve. The AUC value was calculated to obtain the accuracy by which the model predicts gastric cancer T staging.

## Results

### Evaluation of the Training Effects of the Faster RCNN Operating System

**Figure 4:**
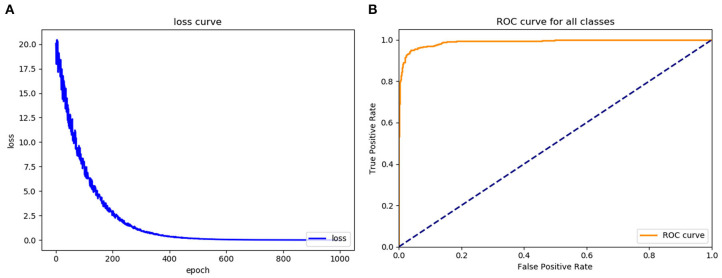
Assess the learning effects of the Faster RCNN operating system. **(A)** LOSS learning curve of the experimental system, **(B)** the ROC curve of the automatic recognition model for advanced gastric cancer recognition had AUC of 0.93.

To assess the learning effects of the Faster RCNN deep neural network, we input the training set into the trained Faster RCNN. The training loss result for the learning curve of the diagnostic platform suggested that the diagnostic platform achieved optimal optimization parameters after 600 learning epochs (Figure 4A). The AUC of the ROC curve for the Faster RCNN operating system in identifying gastric cancer tumors was 0.93, with an accuracy of 0.93 and a specificity of 0.95 comparable to that of the radiologists (Figure 4B).

**Figure 5:**
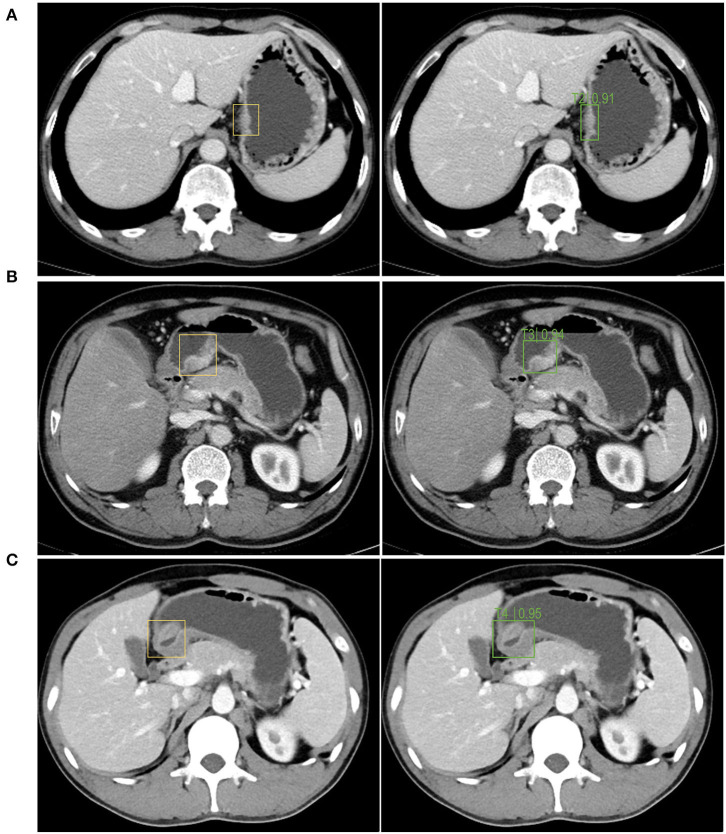
The autorecognition model recognizes the T2 **(A)**, T3 **(B)**, T4 **(C)** stage of the tumor in the image after segmenting and identifying the tumor. The left side of figures were the images wherein the physician manually identified the tumor position based on the pathological results for the training and testing of the model. The right side were the segmentation of the tumor and the identification of the T stage by the recognition model.

The left side of Figure 5 is the image wherein the physician manually identified tumor position based on the pathological results for the training and testing of the model. The right side is the segmentation of the tumor and the identification of the T stage by the recognition model. As shown in Figure 5, the accuracy of the T2, T3, and T4 stage identified by the trained Faster RCNN approach were 0.91, 0.94, and 0.95. It can be concluded that the operating platform has high accuracy in recognizing the gastric cancer T stage based on enhanced CT images. Therefore, Faster RCNN operating system had been effectively trained for images of T stage in gastric cancer.

### Clinical Validation of the Diagnosis of T Stage in Gastric Cancer by the Artificial Intelligence System

**Figure 6:**
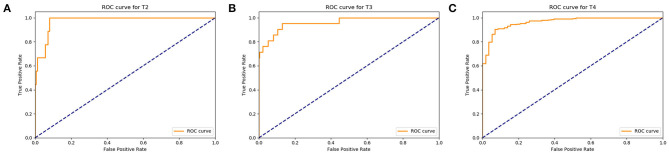
Assess the diagnosis effects of the Faster RCNN operating system. **(A)** The ROC curve of the automatic recognition model for T2 stage gastric cancer recognition had AUC of 0.90, **(B)** the ROC curve of the automatic recognition model for T3 stage gastric cancer recognition had AUC of 0.93, **(C)** the ROC curve of the automatic recognition model for T4 stage gastric cancer recognition had AUC of 0.95.

After the validation were completed, the recognition rate of T2 stage gastric cancer was 90%, the recognition rate of T3 stage gastric cancer was 93%, and the recognition rate of T4 stage gastric cancer was 95% (Figure 6). The results of classification-report in the Metric module were shown in Table 3. It can be concluded that the automatic recognition model has high recognition performance for gastric cancer.

**Table 3:** Classification-report in the Metric module.

	**Precision**	**Recall**	***F*1-score**
T2	0.90	0.87	0.89
T3	0.93	0.91	0.92
T4	0.95	1.00	0.96
Micro average	0.93	0.93	0.93
Macro average	0.94	0.92	0.91
Weighted average	0.93	0.93	0.92

## Discussion

Gastric cancer T staging is an important basis to guide the treatment of gastric cancer. The eighth edition of the National Comprehensive Cancer Network (NCCN) guidelines proposed that upper abdominal enhanced CT is the main diagnostic imaging method for gastric cancer T staging. Patients with early-stage gastric cancer can be treated with endoscopic or surgical resection; patients with T3 and above gastric cancer should be treated first with neoadjuvant therapy and then surgical resection. Accurate preoperative staging is critical for selecting perioperative treatment options and for predicting the prognosis of patients with gastric cancer (12, 14, 15). Radiologists mainly determine the tumor–node–metastasis (TNM) stage of gastric cancer based on preoperative abdominal CT and other imaging data, after which a treatment plan is selected. However, in current clinical practice, there are still some issues regarding TNM staging by preoperative abdominal CT. Different radiologists may determine different TNM stages based on the same abdominal CT data. In the context of the large patient population cared for by three-tier hospitals in China, radiologists have tremendous workloads while being challenged by the complexity of clinical staging of gastric cancer through CT and other imaging data. Staging by doctors is a subjective assessment, to a certain extent, and lacks objectivity. Therefore, a new approach is needed to strike a balance between the increasing workload of radiologists and the need for efficient clinical diagnoses.

The development of deep learning network technology has provided a possible solution to these problems. A CNN computer-aided diagnosis (CAD) system has been applied to the classification and detection of breast histopathological images (16) and colorectal cancer detection (17). Early studies from this research group developed an automatic magnetic resonance image recognition system for metastatic lymph nodes in rectal cancer based on a deep learning network (3, 17, 18). Based on the above research experience, a T stage diagnostic platform for advanced gastric cancer based on a CNN was established, and its clinical value was evaluated.

In this study, enhanced CT images labeled by senior radiologists were used to train the T stage diagnostic platform for advanced gastric cancer, and the results were verified using clinical pathological section results. The diagnosis based on continuous venous phase images of gastric cancer was consistent with the T staging based on the postoperative pathological results. The area under the ROC curve for the diagnostic platform was 0.93, and the accuracy rates for T2, T3, and T4 gastric cancer were 90, 93, and 95%, respectively, which were close to the diagnostic levels of the senior radiologists. The results of this experiment can be explained as follows. T4 tumors are relatively larger than T2 and T3 tumors and infiltrate the serosal layer; therefore, they are easily identified in CT images. In contrast, T2 and T3 tumor invasion affects the submucosa and muscles of the stomach wall, respectively, and these two layers together constitute a low-density striped layer under enhanced CT. Given that gastric cancer, infiltration is accompanied by changes in inflammation and edema, the accurate identification of the gastric cancer T stage is challenging, leading to ambiguous conclusions. There were more images of T4 tumors, and the parameters for the diagnostic platform were optimized better. There were fewer images of T2 and T3 gastric tumors than there were of T4 gastric tumors, and the optimization of the relevant parameters of the diagnostic platform was relatively poorer. The final T stage determinations made by the diagnostic platform based on continuous venous phase images were completely consistent with the postoperative pathological T stage diagnosis. These results suggest that the diagnostic platform has high feasibility, accuracy, objectivity, and efficiency. It is expected that the developed platform will assist radiologists during screening and reduce their workload. Furthermore, the platform will help guide clinicians in determining diagnoses and developing treatment plans and enable patients with gastric cancer to receive more precise and personalized treatment.

The limitations of this study are as follows. This study was a single-center trial with limited data. The artificial intelligence platform constructed in this study can reliably determine T stages, but the T stage evaluation accuracy for single images is not ideal. Increased data volume and algorithm optimization are needed to improve the diagnostic performance. This study is based on the supervised learning of a CNN. The training accuracy of the platform depends on accurate tumor area identification in enhanced CT images by radiologists. We aim to develop and utilize highly efficient data labeling methods with weak supervision or using unsupervised techniques. We explored the feasibility of applying deep learning in this study, and it is necessary to further verify the clinical application value of this diagnostic platform in clinical practice. Therefore, to further improve the reliability of the artificial intelligence-assisted platform, the volume of data will need to be increased using a multicenter approach, and the algorithm and the labeling efficiency will need to be optimized; with those improvements, clinical verification will be realized to achieve the goal of assisting clinicians in diagnosis and treatment.

## Conclusion

This study show that the convolutional neural network computer-aided system established in this study can automatically segment and recognize T stages based on enhanced CT images of advanced gastric cancer, with an accuracy comparable to that of experienced radiologists. The platform is expected to assist radiologists in making more accurate, intuitive, and efficient diagnoses, greatly reducing the workload of radiologists, guiding clinicians in determining diagnoses and developing treatment plans, and helping patients receive more precise and personalized treatment.

## Data Availability Statement

The datasets generated for this study are available on request to the corresponding author.

## Ethics Statement

The studies involving human participants were reviewed and approved by ethics committee of Qingdao University affiliated Hospital. The patients/participants provided their written informed consent to participate in this study.

## Author Contributions

DW and YL: study concept and design. JH, YG, and XiaoZ: acquisition of data. XiZ, MZ, and TN: analysis and interpretation of data. LZ, XuZ, and DW: critical revision of the manuscript and obtained funding. LZ and XuZ: statistical analysis. All authors contributed to the article and approved the submitted version.

## Conflict of Interest

The authors declare that the research was conducted in the absence of any commercial or financial relationships that could be construed as a potential conflict of interest.
