# Supplementary material for: Establishment and Applicability of a Diagnostic System for Advanced Gastric Cancer T Staging Based on a Faster Region-Based Convolutional Neural Network
Source: Front Oncol. 2020 Jul 28;10:1238. doi: 10.3389/fonc.2020.01238 (PMC7399625; doi:10.3389/fonc.2020.01238)
Supplement: Supplementary file 1 [file Data_Sheet_1.docx]

**Supplementary Methods**

**Automatic identification of gastric cancer based on deep learning**

This study adopted the standard Faster R-CNN architecture, which consists of three parts: (1) a feature extraction network, a region proposal network (RPN), and a proposal classiﬁcation and regression network. First, the feature extraction network used the Resnet 50 network to generate a convolutional feature map of the gastric cancer image. Then, the feature map was input into the RPN to generate candidates (also called regions of interest [ROIs])(1) We added a small network for sliding scanning to the convolutional feature map. This network was fully connected to a 3 × 3 sliding window. Then, the sliding window was mapped to a 256-dimensional feature vector. We put this 256-dimensional feature vector into two sliding 1 × 1 fully connected layers, one for the bounding box regression to obtain the coordinates of the proposals and the other for the proposal classiﬁcation to predict the probability score. At each sliding window, multiple region proposals were simultaneously predicted. The proposals were parameterized to some reference boxes, which we called anchors. If the number of anchors for each sliding position is denoted as k, the regression layer has 4k outputs encoding the coordinates of k bounding boxes, and the classiﬁcation layer outputs 2k probability scores that predict the probability of each proposal being an object or not. An anchor is at the center of the indicated sliding window and is related to the scale and aspect ratio. In our experiment, we used three scales and three aspect ratios, which generated nine anchors (k = 9) at each sliding position. To acquire the candidate region proposals, a binary class label (being an object or not) was assigned to each anchor. A positive label was assigned to two types of anchors: (1) an anchor that has the highest intersection-over union (IoU) overlap with a ground truth bounding box or (2) an anchor that has an IoU overlap higher than 0.7. If the IoU of an anchor was less than 0.3 with all the ground truth boxes, then a negative label was assigned to the anchor. Using this method, the candidate regions, which are potential sites for gastric cancer, were generated on a convolutional feature map. Then, non-maximum sup pression was adopted to merge neighboring regions to reduce the number of region proposals for training, which substantially reduced the redundant calculations for proposal regression and classiﬁcation(2). The convolution map shared by the RPN and feature extraction network can acquire the coordinates and class probability scores of the predicted bounding boxs using the ROI pooling layer and the two succeeding sibling fully connected layers(3).In this study, alternating training was performed by calculating the regression loss of the predicted bounding boxes compared to the ground truth boxes and the classiﬁcation loss of the proposals. We trained the network through back propagation and stochastic gradient descent (SGD), and the network weight and parameters can be continuously updated and optimized. Finally, we obtained the ﬁnal model as the AI diagnosis system.

**References:**

1. Shin HC, Roth HR, Gao M, Lu L, Xu Z, Nogues I, et al. Deep Convolutional Neural Networks for Computer-Aided Detection: CNN Architectures, Dataset Characteristics and Transfer Learning. *Ieee T Med Imaging* (2016) 35(5):1285-98.

2. He K, Zhang X, Ren S, Sun J. Spatial Pyramid Pooling in Deep Convolutional Networks for Visual Recognition. *Pattern Analysis & Machine Intelligence IEEE Transactions on* (2015) 37(9):1904-16.

3. Ren S, He K, Girshick R, Sun J. Faster R-CNN: Towards Real-Time Object Detection with Region Proposal Networks. *IEEE Transactions on Pattern Analysis & Machine Intelligence* (2017) 39(6):1137-49.
